# Profile of Brazilian Chia Consumers: Knowledge of Health Benefits and Recipes for Consumption

**DOI:** 10.1007/s11130-026-01529-6

**Published:** 2026-06-19

**Authors:** Juliana Delvizio, Lilia Zago, Flávia Oliveira Brito, Mayara de Jesus Santos Moura, Flávia dos Santos Barbosa Brito, Carolina Beres, Roberta Fontanive Miyahira

**Affiliations:** Graduation Program in Food, Nutrition and Health, Institute of Nutrition, State University of Rio de Janeiro, Rua São Francisco Xavier, 524, 12◦ andar, sala 12006 D – Maracanã, Rio de Janeiro, RJ CEP: 20550-013 Brazil

**Keywords:** Chia, Chia-based products, Consumer behavior, Consumer preference, Consumer education, Nutrition

## Abstract

Chia is recognized for its important nutritional and technological properties. Chia seeds are currently grown in various regions around the world, including Brazil. However, studies evaluating chia consumption are still scarce. This study aimed to investigate the characteristics of chia consumers in Brazil, including their knowledge of the seed’s properties, consumption habits, and culinary uses. A self-administered questionnaire was used. A total of 747 individuals participated in the survey, with 468 reporting consuming chia and 279 reporting not consuming chia. Females (67.1%) and individuals with a high level of education (67.6%) were the groups most likely to report consuming chia. The word “fibre” was the term most frequently associated with chia by the participants. Most participants reported consuming chia daily (23.7%) or weekly (39.7%). Chia was mostly incorporated in yoghurt (*n* = 313), fruit (*n* = 301), and smoothies (*n* = 160). Improvement in bowel function, increased satiety, and contribution to weight loss were the most cited health benefits associated with chia consumption. Non-consumption of chia was mainly related to lack of habit (31.5%), aversion to the taste (30.8%), or lack of familiarity with the product (7.5%). However, non-consumers indicated that they would be interested in consuming chia if they were aware of its health benefits. These findings highlight the importance of consumer education and clear communication of health information to promote the chia consumption. The study suggests that developing chia-based or chia-enriched products can combine health benefits with taste, convenience, and accessibility.

**Supplementary Information:**

The online version contains supplementary material available at https://doi.org/10.1007/s11130-026-01529-6.

## Introduction

Chia (*Salvia hispanica* L.) is a herbaceous plant belonging to the *Lamiaceae* family and the *Salvia* genus, with about 900 species distributed across various regions of the world [1]. The seeds, ranging in size from 1 to 2 mm, have a smooth shell and variable colouration, including shades of black, white, and brown [2]. Native to southern Mexico and northern Guatemala, chia is currently grown in countries such as Australia, Bolivia, Colombia, Brazil, Peru, and Argentina, with Mexico being the world’s largest producer [3]. It is preferably grown in tropical to subtropical climates, and its chemical composition can vary significantly depending on environmental factors such as climate, altitude, soil type, and harvest season [4]. Recent studies have suggested that colder and higher-altitude environments tend to produce seeds with higher levels of nutrients, such as omega-3 fatty acids [1, 4]. In Brazil, cultivation is more concentrated in the southern region, especially in the states of Paraná and Rio Grande do Sul, where milder climatic conditions and higher altitudes favour its development compared to other regions of the country [5].

Chia seeds have a significant nutritional composition, with approximately 38 g of carbohydrates, 17 g of protein, and 33 g of lipids *per* 100 g, values that exceed those of other cereals such as rice, wheat, and corn and are comparable to or even exceed those of legumes in terms of protein content [6]. Their lipid profile is characterised by a predominance of polyunsaturated fatty acids, especially α-linolenic acid (omega-3), which accounts for approximately 60% of the total, followed by linoleic acid (omega-6), oleic acid (omega-9), and small amounts of saturated fatty acids such as palmitic acid [7]. The seed is also rich in dietary fibre (35 g/100 g), which is mostly insoluble, while the soluble fraction is composed mainly of mucilage, capable of absorbing up to 27 times its weight in water and forming a gel [8]. In addition, chia contains minerals, including calcium, phosphorus, potassium, magnesium, iron, and zinc, as well as bioactive compounds and phytosterols with antioxidant properties [9]. The components of chia seeds play an important role in preventing chronic disease [10]. Studies conducted in humans and animals have linked chia seeds to improvements in insulin resistance, abnormal lipid profiles, glucose tolerance, and weight loss [11, 12].

Besides its nutritional value, chia has been widely studied for its technological applications in industry and gastronomy [13]. Due to a strong water absorption capacity, the gels formed by chia function as a natural substitute for fats and eggs in culinary preparations, maintaining good sensory characteristics [7]. The versatility of the seed is reflected in its use in various food products, such as breads, pasta, cookies, cakes, and sauces such as mayonnaise, which increases the nutritional value of the product by raising the content of proteins, minerals, and polyunsaturated fatty acids [13].

Chia is being used not only by industry but also in home cooking, expanding its consumption possibilities. Thus, chia is establishing itself not only as a functional ingredient but also as a versatile technological resource in cooking. Despite its nutritional and technological advantages, only one study evaluating the effects of chia seed consumption was found in the scientific literature [14]. Understanding the factors that influence food choices can help encourage consumption and promote the selection of foods beneficial to the population’s health, especially for foods not part of the local culture, as with chia in Brazil. Thus, the objective of this study was to evaluate the profile of chia consumers in Brazil, analyzing their knowledge about the properties of the seed, as well as consumption practices and culinary applications.

## Materials and Methods

A non-probability snowball sampling method was used for data collection. In this method, study participants recruit additional individuals from among their contacts until sufficient data is obtained [15]. The sample size was determined using the formula described by Daniel and Cross [16], assuming a significance level (⍺) of 5% (*i.e*., 95% confidence level). The margin of error was established at 3.6%, with the estimated proportion at 50%, reflecting the most conservative estimate and providing the highest sample size [12]. The calculation provided a sample of 742 individuals. The participants (*n* = 747), all over the age of 18, were recruited through social networks and participated in the study on a voluntary basis, based on their interests and availability.

The data collection instrument was a self-administered questionnaire, developed based on the study by Gonçalves et al. [17], made available on the Google Forms^®^ platform between September 2024 and April 2025. Participation was voluntary, anonymous, and conditional on the acceptance of the Free and Informed Consent Form. The project was approved by the Research Ethics Committee of the Pedro Ernesto University Hospital of the State University of Rio de Janeiro (CAAE 50519321.3.0000.5259, opinion 7.072.799).

The questionnaire consisted of 14 to 18 questions (Fig. S1). Different types of questions were used: closed single-choice, closed multiple-choice, and open-ended. The initial section of the questionnaire sought to outline the sociodemographic profile of the respondents, including information on gender, age group, educational level, profession, monthly family income, city of origin, and current place of residence. The open-ended responses regarding occupation were subsequently categorised according to the Brazilian Classification of Occupations [18].

Participants were also asked to report their dietary patterns, being classified as omnivores, ovo-lacto vegetarians, vegetarians, vegans, or raw foodists. The second part of the questionnaire focused on assessing knowledge and habits related to chia consumption. Questions were included on the usual form and frequency of consumption, the foods with which chia is usually combined, places of purchase, motivations for purchase, perceptions of nutritional properties and health benefits, as well as the first two words participants associate with the food. Questions about chia seeds’ culinary uses and health benefits could have multiple-choice options. Participants who reported not consuming chia were automatically redirected to perception questions, ensuring their partial inclusion in the qualitative analyses. Participants who reported consuming chia also specified the most commonly used form, and the most frequent modes of consumption. They were also asked about their usual places of purchase and the main reasons that influenced them to buy chia, such as nutritional value, taste, or association with healthy eating.

Quantitative data were organized in Excel^®^ spreadsheets and descriptively summarized in tables. Sociodemographic characteristics and eating habits were described using absolute and relative frequencies according to chia consumption status, and differences between groups were assessed using the Chi-square test. The qualitative session, which addressed the open question “What are the first two words that come to mind when you think of chia?”, was conducted using MAXQDA Analytics Pro (version 24.11.0). After importing the texts, the data was cleaned, excluding terms of low analytical relevance, such as articles, prepositions, and pronouns. The Word Cloud tool was used [19].

## Results and Discussion

Online questionnaires are widely recognized as effective tools for investigating opinions and behaviours across different population groups. In the present study, the questionnaire was completed by 747 individuals, of whom 468 reported consuming chia and 279 did not. Table 1 presents the sociodemographic characteristics separated by chia consumers and non-consumers, including gender, age group, marital status, educational level, family income, region, and eating habits profile. In both groups, the majority of respondents were female, aged between 18 and 35 years, with a postgraduate level of education and a household income between 2 and 5 Brazilian minimum wages. Regarding marital status, the majority of chia consumers were married, and non-chia consumers were single. In addition, most of the participants reported omnivorous eating habits in both groups. It should be noted that previous studies conducted with online questionnaires have shown that women are generally more likely to respond to questionnaires and engage in research activities [20, 21], which may explain the significant number of female participants in both groups. In addition, the predominant age group in both groups (> 50%) was 18–35 years old. Young adults are in the process of establishing lifestyle habits, including eating habits, making them an attractive group for functional food consumption [22, 23]. Table 1 also shows that the proportions of gender, age group, marital status, education level, and region were statistically different (*p* ≤ 0.05) between the groups of chia consumers and non-consumers. However, there was no statistical difference (*p* > 0.05) in the proportion of family income and eating habits profile between the two groups. Occupations were collected through an open question, allowing participants to describe their profession in their words. The analysis showed that the majority (approximately 54%) were professors, students, and nutritionists. The second most frequent group comprised health-related professionals (11%).

**Table 1:** Sociodemographic characterization and eating habits profile of chia consumers (*n* = 468) and non-consumers (*n* = 279)

Variable	Chia consumption								*P*-value*
	Yes		95% CI		No		95% CI		
	n	%			n	%			
Gender^a^									
Female	401	67.1	63.2	70.7	197	32.9	29.3	36.8	0.01
Male	66	44.9	37.0	53.0	81	55.1	47.0	63.0	
Age									
18-25 years old	104	59.1	51.7	66.1	72	40.9	33.9	48.3	0.05
26-35 years old	122	61.0	54.1	67.4	78	39.0	32.7	46.2	
36-45 years old	107	70.9	63.1	67.5	44	29.1	22.0	36.4	
46-55 years old	71	67.6	58.1	75.9	34	32.4	23.5	41.3	
> 55 years old	64	55.7	46.5	64.5	51	44.4	35.0	53.1	
Marital Status^b^									
Single	215	57.2	52.0	62.0	161	42.8	38.0	48.0	0.01
Stable Union / Married	223	69.9	64.6	74.7	96	30.1	25.3	35.4	
Divorced	27	65.9	50.3	78.6	14	34.1	21.4	49.7	
Widowed	3	37.5	12.5	71.6	5	62.5	28.4	87.5	
Education level^c^									
Incomplete high school	0	0	-	-	2	100	-	-	0.01
Complete high school	85	52.5	44.8	60.1	77	47.5	40.0	55.2	
Complete graduation	130	62.5	55.7	68.8	78	37.5	31.2	44.3	
Post-graduation	253	67.6	63.0	72.5	121	32.4	27.5	37.0	
Remuneration (Brazilian minimum wage)^d^									
Until 1	25	58.1	43.1	71.8	18	41.9	28.2	56.9	0.11
2 to 5	181	61.2	55.5	66.5	115	39.0	33.6	44.7	
6 to 9	88	59.1	51.0	66.7	62	40.9	33.7	49.4	
More than 10	139	69.2	62.2	75.2	62	30.9	24.2	36.9	
Region									
North	2	100	-	-	0	0	-	-	0.05
Northeast	17	56.7	38.8	72.9	13	43.3	27.1	61.2	
Southeast	406	61.8	58.0	65.4	251	38.2	34.6	42.0	
South	27	65.9	50.3	78.6	14	34.1	21.4	49.7	
Midwest	16	94.1	67.9	99.2	1	5.9	0.8	32.1	
Eating habits									
Omnivore	426	61.8	58.1	65.4	263	38.2	34.6	41.9	0.12
Ovolactovegetarian	35	72.9	58.7	83.6	13	27.1	16.3	41.3	
Vegan	5	100	-	-	0	0	-	-	
Vegetarian	2	50.0	12.3	87.7	2	50.0	12.3	87.7	
Crudivore	0	0	-	-	1	100	-	-	

*CI*, Confidence interval; * *P*-value obtained by the Chi-square test

^a^Two individuals who preferred not to disclose their gender were excluded

^b^Three individuals who preferred not to disclose their marital status were excluded

^c^One individual who preferred not to disclose their educational level was excluded

^d^Fifty-seven individuals who preferred not to disclose their family income were excluded

The only study that evaluated the effects of chia seed consumption was conducted in India, and most of the urban respondents in the survey were female, aged 20–50, and had a PhD level of education [14]. Flaxseed has a nutritional profile similar to chia, a seed also classified as a functional food, and its consumption has been studied in Brazil. The study included a sample of 395 respondents, predominantly aged 20 to 49 years (73%), with the majority being female (65%), single (55%), and students (37%). In addition, 30% had not completed higher education, and 44% reported a household income between 1 and 3 minimum wages [24].

**Fig. 1:**
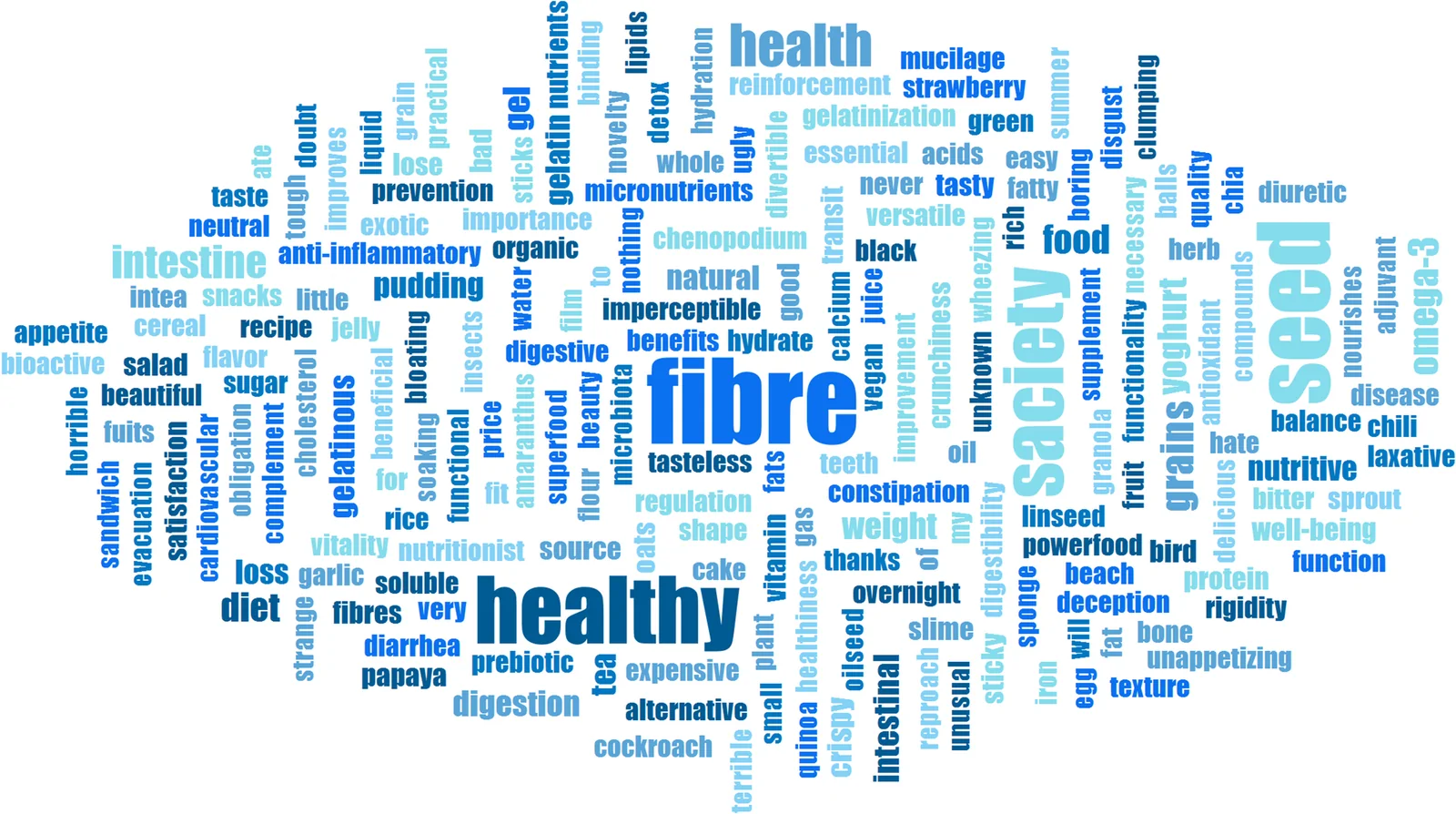
Word cloud highlighting the words cited by study participants

Word association (WA) is an effective, quick, and easy-to-use method for obtaining information about consumer perceptions of a product [22]. A total of 200 words were mentioned. Based on word association, a word cloud was created (Fig. 1), highlighting the most frequently cited words, including “fibre”, “seed”, “satiety”, and “healthy”. These terms indicate a positive perception of chia, highlighting its nutritional benefits. Other terms mentioned, such as “yoghurt”, “pudding”, and “gelatina”, point to common ways of consuming and using the seed in cooking. Thus, the word cloud expressed both popular recognition of chia’s functional properties and its application in daily eating habits.

Most study participants who consumed chia indicated a preference for consuming the seed (89%), and only a small portion (2.6%) consumed chia flour (Table S1) exclusively. Despite the high nutritional value of chia seeds, during gastrointestinal digestion, the nutrients in whole seeds may not be released from the food matrix and become available for absorption [25]. Calvo-Lerma et al. [25] demonstrated that grinding chia seeds has a positive impact on the digestibility of macronutrients and the bioaccessibility of calcium and polyphenols. After the simulated in vitro digestion process, ground chia seeds had higher bioaccessible calcium (4.22 mg/g) and polyphenol (6.13 mg GA eq./g) values than whole chia seeds (3.82 mg/g and 1.81 mg GA eq./g, respectively). In addition, the digestibility of lipids and proteins was higher when the seed was crushed [25]. There is still a lack of knowledge about the best way to consume chia seeds, and further studies on the subject and greater dissemination of the results are necessary so that chia consumption can provide greater benefits to human health.

In the present study, most participants (> 60%) reported consuming chia daily or weekly (Table S1). The main place of purchase for chia was convenience stores, followed by supermarkets and grocery stores, demonstrating that chia is widely available (Table S1). The main reasons cited for chia consumption were that it is a nutritious or healthy food or that it was recommended by a nutritionist (Table S1). Regarding the health benefits related to chia consumption, consumers most frequently cited that it aids intestinal function (*n* = 605), increases satiety (*n* = 561), aids weight loss (*n* = 374), helps reduce cholesterol (*n* = 357) and blood sugar (*n* = 285), boosts immunity (*n* = 145), aids blood pressure control (*n* = 144), and has anti-inflammatory (*n* = 269) and antioxidant properties (*n* = 345). Studies show that regular consumption of chia can bring benefits to human health, since chia is considered a functional food due to its high content of omega-3 fatty acids and phytochemicals that have antioxidant action [9, 26, 27]. Chia is also linked to lower blood triglyceride levels and blood pressure, as well as increased satiety after meals [28].

The study on chia seed consumption among the urban population in India revealed that the main reason for consumer preference was the abundant presence of polyunsaturated fatty acids (PUFAs), the association with the prevention of heart disease, anti-inflammatory, and antioxidant properties [14]. A study on flaxseed consumption conducted in Brazil reported improved bowel regularity (41%) and reduced cholesterol levels (16%) as health benefits [24].

Adding chia to culinary preparations and products has nutritional and technological benefits [29]. Participants reported that the main ways of consuming the seed were by adding it to yoghurt or fruit. Consumption in baked goods was quite common, with bread, pancakes, and cakes being the most frequently mentioned. The study evaluating urban chia consumers in India found that participants reported preferring to consume chia in snacks (92%), beverages (83%), and pudding (67%) [14].

The inclusion of chia in baked goods has been the subject of many studies aimed at evaluating the impact of adding chia seeds on improving the nutritional value and acceptance of these products. The results indicated that adding this seed to preparations can increase the levels of protein, omega-3 fatty acids, dietary fibre, and total phenolic compounds [30–32]. In addition to nutritional characteristics, chia-containing food products have also demonstrated good sensory acceptance, a challenging factor in food product development that cannot be ignored. In the study by Borges et al. [30], the addition of chia seeds (6%) or flour (4%) in the development of gluten-free bread was well accepted by consumers, with overall acceptance around 70%. This value is in line with the minimum percentage required for the product to be accepted in the market [33].

The study by Sharma et al. [31] also revealed good acceptance in muffin formulations with different amounts of chia seed (5%; 10%; 15%; 20%; 25%). The authors observed greater acceptance of the product as the addition of chia seeds to the muffin increased. According to the authors, this may have occurred due to the production of chia mucilage, which increases the product’s water content and reduces the sensation of dryness [31]. The use of chia in the development of vegan and plant-based products further highlighted the seed’s multifunctionality [29]. Moreira et al. [32] developed a vegan brownie by replacing eggs with chia mucilage. The vegan brownie had an acceptability rating of 76%, meaning that participants liked the preparation. Most evaluators stated that they would likely purchase the vegan formulation, noting its excellent appearance and a soft texture similar to that of a traditional cake.

In the present study, chia consumption in beverages such as smoothies, juices, and milkshakes accounted for 42% of the responses. Paramita et al. [34] observed that adding chia seeds to smoothies increased the nutritional content of this beverage. The smoothie formulations contained carrots, strawberries, apples, bananas, orange juice, sugar, and chia seeds in different amounts (0, 2.5, 5, or 7.5 g). Even with the addition of only 2.5 g of chia, changes in the composition of the smoothie were observed, increasing the protein, lipid, and dietary fibre content. A previous study showed that chia seeds contain excellent protein and lipid values, representing about 19% and 40%, respectively [27]. Chia also contains all the essential amino acids for human nutrition, including isoleucine, leucine, methionine, phenylalanine, lysine, threonine, histidine, tryptophan, and valine [8]. Furthermore, in the present study, the consumption of chia in salads (*n* = 144), overnight (*n* = 135), granola (*n* = 106), and omelettes (*n* = 95) was frequently mentioned. Finally, jam (*n* = 44), pudding (*n* = 14), and mousse (*n* = 10) were also mentioned as ways to consume chia. When in contact with water, chia seeds develop a mucilage around them. This mucilage can be advantageous in various food preparations, such as pudding, jam, and mousse, as it has the potential to be used as a thickener, emulsifier, and stabilizer [35].

Approximately 37% of participants reported not consuming chia seeds (Table S2). The most frequently cited reasons were “lack of habit” (*n* = 88), followed by “I don’t like it” (*n* = 86). A total of 21 people reported not knowing this food. The options “the price is not affordable” (*n* = 18), “difficulty finding it” (*n* = 16) and “I don’t see any benefit” (*n* = 9) were also cited. This result showed that chia is relatively unknown among and not part of the eating habits of the Brazilian population. However, about 78% of these participants reported they would consume chia seeds if they knew it would have beneficial effects. Miyahira et al. [22], who evaluated consumers’ perceptions of edible sprouts, also noted that most participants would likely be interested in purchasing sprout products if they knew about their high nutritional value. However, in the study on consumer behaviour regarding flaxseed in Brazil, it was observed that although they were aware of the health benefits of consuming flaxseed, the majority of respondents (58%) did not use products containing the seed due to a lack of interest and curiosity [24].

## Conclusions and Future Perspectives

This study provides useful insights into the profile, perceptions, and consumption patterns of chia in Brazil, based on an online survey. Female participants with a high level of education were more likely to consume chia, mainly due to claims of its health benefits. Higher consumption of whole chia seeds was observed when added to foods such as yoghurt, fruit, smoothies, and salads. This indicated that chia can be easily incorporated into the daily diet. On the other hand, non-consumption of the product was mainly related to a lack of habit, aversion to the taste, or unfamiliarity with it. However, many people who did not consume chia said they would be interested in it if they knew its health benefits. These results highlight the importance of educating consumers and communicating health information clearly to encourage the consumption of functional foods, such as chia. This study has some limitations, including its cross-sectional design, which limits causal inferences; the results may be affected by memory bias, as participants may recall or report their consumption habits inaccurately, and by self-selection bias, since those who choose to participate may differ systematically from the general population. Finally, these findings indicate that knowledge about the perception of chia consumption habits could be useful as a basis for future studies on product development that focus on exploring strategies to enhance sensory acceptance and promote the incorporation of chia into foods frequently consumed by different demographic groups.

## Supplementary Information

Below is the link to the electronic supplementary material.

Supplementary Material 1

## Data Availability

Data will be made available on request.
