# Supplementary material for: Profile of Brazilian Chia Consumers: Knowledge of Health Benefits and Recipes for Consumption
Source: Plant Foods Hum Nutr. 2026 Jun 19;81(3):79. doi: 10.1007/s11130-026-01529-6 (PMC13282295; doi:10.1007/s11130-026-01529-6)
Supplement: Supplementary file 1 — Supplementary Material 1 [file 11130_2026_1529_MOESM1_ESM.docx]

**What do Brazilian consumers know about health benefits of chia and how can it be incorporated into their diets?**

**Supplementary material**

**Fig S1. Questionnaire about chia consumption in Brazil**

1) Do you agree to participate in this study?

o Yes

o No (you will be redirected to the completion page)

2) Email: ________________________

3) Which gender do you identify with:

o Female

o Male

o I prefer not to say

4) Age:

o 18–25 years old

o 26–35 years old

o 36–45 years old

o 46–55 years old

o > 55 years old

5) Marital status:

- Single
- Stable Union / Married
- Divorced
- Widowed
- Prefer not to say

6) What is the highest level of education you have completed?

- Incomplete High school
- Complete High school
- Complete graduation
- Post-graduation
- I prefer not to say

7) What is your approximate household income?

o Up to 1 minimum wage

o 2–5 minimum wages

o 6–9 minimum wages

o More than 10 minimum wages

o I prefer not to say

8) What is your occupation? ____________

9) Current state of residence: __________

10) Regarding your eating habits, do you consider yourself?

o Omnivore (eats animal and plant-based foods without restriction)

o Ovo-lacto-vegetarian (eats plant-based foods, eggs, milk, and cheese)

o Vegetarian (eats only plant-based foods)

o Vegan (consumes plant-based foods and foods not produced by animals)

o Raw foodist (eats only raw plant-based foods)

11) What are the first two words that come to mind when you think of chia? (insert an image of chia)

12) Do you consume chia? If so, how often?

o Daily

o Weekly

o Twice a month

o Monthly

o Annually

o I do not consume it **

13) If “I do not consume it,” the participant should select the reason(s) below:

- I am not familiar with this food
- Lack of habit
- The price is not affordable
- I don’t like it (the appearance, texture, taste, etc.)
- Difficulty finding it where I usually shop or eat
- I don't see any nutritional benefits
- Other reason(s)_______

(go to question 18)

14) In what form do you consume chia?

o Seeds

o Flour

o Seeds and flour

15) How do you consume chia?

o On fruit

o With yoghurt

o In soup

o In juice

o In salad

o On foods

o Pudding

o Cookies

o Saltines crakers

o Cakes in general

o Pies in general

o Bread in general

o Sprouts

o Smoothies

o Pancakes

o Overnight

o Ice cream

o Jelly

o Brigadeiro

o Omeletts

o Milkshakes

o Sauces

o Mousse

o Cheese bread

o Granola

o Brownie

o Others__________

16) Where do you usually buy chia?

o Supermarkets

o Grocery stores

o Produce markets

o Health food stores

o Convenience stores

o Directly from producers

o Online

o Other _____

17) Why do you consume chia (check all options you consider appropriate)?

o It is a nutritious food

o It is a healthy food

o It is a tasty food

o Recommended by a nutritionist

o Recommended by a doctor or other healthcare professional

o Due to media influence

o Other _____

18) Which of the following properties do you think chia has (check all options you think are correct)? **

o Antioxidant action

o Anti-inflammatory action

o Reduces blood cholesterol

o Reduces blood sugar

o Increases immunity

o Increases satiety

o Aids intestinal function

o Aids weight loss

o Aids blood pressure control

o Other: _______

o I don’t know

19) Chia consumption is associated with health benefits. Knowing this, would you consume chia or a product made with chia?

- Yes, definitely
- Yes, probably
- No, definitely
- No, probably
- I don't know

** Respondents who answered “I do not consume” to question 13 should be directed to question 18

**Table S1. Response from chia consumers regarding form and frequency of consumption, place of purchase, and reasons for chia consumption**

| **Question** | | **n** | **Frequency (%)** | **95%CI** | |
| --- | --- | --- | --- | --- | --- |
| **How do you consume chia?** | | | | | |
|  | Flour | 12 | 2.6 | 1.4 | 4.5 |
|  | Seeds | 415 | 88.7 | 85.4 | 91.2 |
|  | Seeds and flour | 41 | 8.8 | 6.5 | 11,7 |
| **How often do you consume chia?** | | | | | |
|  | Daily | 111 | 23.7 | 20.1 | 27.8 |
|  | Weekly | 186 | 39.7 | 35,4 | 44.3 |
|  | Twice a month | 55 | 11.8 | 9.1 | 15.0 |
|  | Monthly | 79 | 16.9 | 13.8 | 20.6 |
|  | Annually | 37 | 7.9 | 5.8 | 10.7 |
| **Where do you usually buy chia?** | | | | | |
|  | Convenience stores | 230 | 49.15 | 43.49 | 58.80 |
|  | Supermarkets | 207 | 44.23 | 32.25 | 67.61 |
|  | Grocery stores | 17 | 3.63 | 1.18 | 11.59 |
|  | Other locations | 14 | 2.99 | 1.30 | 11.58 |
| **Why do you consume chia?** | | | | | |
|  | It is a nutritious food | 316 | 67.45 | 47.55 | 121.88 |
|  | It is a healthy food | 100 | 21.35 | 14.05 | 40.39 |
|  | Recommended by a nutritionist | 37 | 7.90 | 5.47 | 13.26 |
|  | Other reason | 15 | 3.17 | 0.70 | 17.53 |
| CI: Confidence interval | |  |  |  |  |

**Table S2. Response regarding reasons for not consuming chia and likelihood of consuming chia after learning about its health benefits**

|  | **Question** | **n** | **Frequency (%)** | **95%CI** | |
| --- | --- | --- | --- | --- | --- |
| **The consumption of chia is linked to beneficial effects on human health. With this in mind, would you consume chia or a product made with chia?** | |  |  |  |  |
|  | Yes, definitely | 112 | 40.14 | 34.53 | 46.03 |
|  | Yes, probably | 105 | 37.63 | 32.12 | 43.49 |
|  | No, definitely | 4 | 1.43 | 0.54 | 3.77 |
|  | No, probably | 24 | 8.60 | 5.82 | 12.53 |
|  | I don't know | 34 | 12.19 | 8.82 | 16.59 |
| **What is the reason for not consuming?** | |  |  |  |  |
|  | Lack of habit | 88 | 31.54 | 16,79 | 94.07 |
|  | I don't like it (the appearance, texture, etc.) | 86 | 30.82 | 24.81 | 42.96 |
|  | I am not familiar with this food | 21 | 7.53 | 4.06 | 22.20 |
|  | The price is not affordable | 18 | 6.45 | 2.60 | 18.05 |
|  | Difficulty finding it where I usually shop or eat | 16 | 5.73 | 3.54 | 9.17 |
|  | I don't see any nutritional benefits | 9 | 3.23 | 2.60 | 18.05 |
|  | Other | 41 | 14.70 | 1.12 | 12.28 |
| CI: Confidence interval | |  |  |  |  |
